# Assessing the Diversity and Stability of Cellular Immunity Generated in Response to the Candidate Live-Attenuated Dengue Virus Vaccine TAK-003

**DOI:** 10.3389/fimmu.2019.01778

**Published:** 2019-07-31

**Authors:** Adam T. Waickman, Heather Friberg, Morgan Gargulak, Amanda Kong, Mark Polhemus, Timothy Endy, Stephen J. Thomas, Richard G. Jarman, Jeffrey R. Currier

**Affiliations:** ^1^Viral Diseases Branch, Walter Reed Army Institute of Research, Silver Spring, MA, United States; ^2^Department of Medicine, Upstate Medical University of New York, Syracuse, NY, United States

**Keywords:** dengue, vaccine, T cell, cellular immunity, epitope discovery

## Abstract

The development of an efficacious DENV vaccine has been a long-standing public health priority. However, this effort has been complicated significantly due to the hazard presented by incomplete humoral immunity in mediating immune enhancement of infection and disease severity. Therefore, there is a significant need for DENV vaccine platforms capable of generating broad immune responses including durable cellular immunity, as well as novel analytical tools to assess the magnitude, diversity, and persistence of vaccine-elicited immunity. In this study, we demonstrate that a single dose of the recombinant, tetravalent, live-attenuated DENV vaccine TAK-003 elicits potent and durable cellular immunity against both the structural and non-structural proteins of all four DENV serotypes, which is maintained for at least 4 months post-immunization. Although not contained within the vaccine formulation, significant reactivity against the non-structural (NS) proteins of DENV-1,-3, and-4 is observed following vaccination, to an extent directly proportional to the magnitude of responses to the corresponding vaccine (DENV-2) components. Distinct, quantifiable, and durable patterns of DENV antigen reactivity can be observed in individuals following vaccination. Detailed epitope mapping of T cell reactivity against the DENV-2 proteome using a matrix of overlapping peptide pools demonstrated that TAK-003 elicits a broad response directed across the DENV-2 proteome, with focused reactivity against NS1 and NS3. We conclude that, as measured by an IFN-γ ELISPOT assay, a single dose of TAK-003 generates potent T cell-mediated immunity which is durable in magnitude and breadth through 4 months post-vaccination.

## Introduction

Dengue virus (DENV) is the causative agent of an acute febrile illness in humans and is a significant source of global morbidity (1). It is an arthropod-borne virus that is transmitted predominantly by the mosquito vectors of the genus *Aedes* (2). DENV is a single-stranded positive sense RNA virus that belongs to the family Flaviviridae, genus *Flavivirus*, and consists of four genetically and immunologically distinct serotypes: DENV-1; DENV-2; DENV-3; and DENV-4. DENV infects between 280 and 550 million people worldwide every year with as many as 100 million infection resulting in clinical presentation (any severity of disease) (1, 3). Infection with DENV is subclinical in the majority of cases, but it may also cause dengue fever, a debilitating flu-like illness that lasts for up to 2 weeks. Approximately 500,000 cases per year develop into severe dengue, dengue hemorrhagic fever/dengue shock syndrome (DHF/DSS), which has a mortality rate of up to 20% (4–7). Dengue is endemic in south and south-east Asia, the western Pacific, sub-Saharan Africa, and Central and South America, and hence at least 40% of the world's population is at risk of infection (1). Extensive globalization of DENV and increases in regional endemic serotype make-up and complexity continue to occur (1). As a result, developing a protective, durable, and safe vaccine product to counter the global threat of DENV infection is a public health priority. However, the difficulty in inducing immunity to all four DENV serotypes, the lack of a validated animal model for vaccine testing, and the lack of defined immune correlates of protective immunity represent significant obstacles to this effort. These challenges are further highlighted by the recent revelation that the only currently available DENV vaccine (Dengvaxia®) not only fails to protect previously DENV naïve individuals from infection, but can increase the risk of hospitalization with virologically confirmed dengue (8, 9). Therefore, there is significant need for new DENV vaccine candidates, as well as new tools and approaches to assess DENV vaccine safety, efficacy, durability, and immunogenicity.

To date, much focus has been placed on measuring neutralizing antibodies (NAb) as an immune correlate of protection against subsequent dengue infection (10–14). However, data from Phase IIb and Phase III trials of the Sanofi-Pasteur vaccine product (Dengvaxia®) demonstrated a discordance between vaccine efficacy and NAb titers (15–17). In addition, while DENV-elicited humoral immunity undoubtedly contributes to protection against homotypic and heterotypic reinfection, the same humoral milieu may play a more pathologic role upon heterotypic infection. While the pathogenesis of DHF/DSS is mechanistically complex and may involve some degree of genetic predisposition (18, 19), waning humoral immunity following infection and partial antibody-mediated cross-recognition of heterotypic DENV is one potential explanation for the increased incidence of severe disease in the setting of secondary infection (5, 20, 21). Antibody-dependent enhancement (ADE) of DENV infection has been demonstrated in numerous *in vitro* experimental models and *in vivo* adoptive transfer animal models (5, 10, 22–24), although these models have not translated to human disease, and definitive *in vivo* evidence of ADE in humans has been elusive.

In light of gaps in knowledge about the relationship between humoral immunity and outcome of DENV infection, there is the need to investigate the contribution of other immune response parameters, particularly cell mediated immunity (CMI)—especially T cell-mediated immunity—to the outcome of DENV infection (25–27). Recent studies suggest that the expression of certain HLA alleles—and the nature/magnitude of the T cell responses they facilitate—correspond to susceptibility or resistance to disease, and potential DENV vaccine efficacy (28, 29). Human T cell responses to DENV were first characterized over 30 years ago, and many of the general principles originally described have remained consistent (25, 27). Infection with one DENV induces both CD4^+^ and CD8^+^ memory T cells specific for DENV epitopes, with a small number of epitopes dominating the response in each individual (28, 29). Epitopes are located throughout the DENV polyprotein, although several regions, especially non-structural protein 3 (NS3) and the capsid protein, appear to have a concentration of immunodominant epitopes targeted by CD8^+^ T cells and CD4^+^ T cells, respectively (30–32). The amino acid homology across the four DENV serotypes varies for each epitope; however, most epitopes are well-conserved among strains within the same serotype and differ at relatively few positions (1–3 of 9 residues) from the corresponding epitopes of other DENV serotypes (and other flaviviruses) (33, 34). As is observed in DENV-elicited humoral immunity, the overall T cell response induced by a primary DENV infection is strongest to the serotype to which the subject was exposed, but variable degrees of cross-reactivity are usually observed to one or more of the other serotypes (35, 36).

TAK-003 is a tetravalent, recombinant DENV vaccine candidate based on the attenuated PDK-53 DENV-2 virus strain that is currently undergoing phase III testing with a two-dose vaccine schedule (37). The PDK-53 strain was initially derived from the WT DENV-2 16,681 isolate, and attenuated by serial passage in primary dog kidney (PDK) cells. Key attenuation mutations have been identified in the 5′ UTR, NS1, and NS3 regions of the viral genome (38). This DENV-2 backbone virus was previously shown to be safe, immunogenic, and capable of stimulating durable cellular and humoral immunity (39–43). To create a vaccine capable of eliciting an immune response against all four DENV serotypes, recombinant viruses were created using the PDK-53 DENV-2 genetic backbone and the prM and E genes from DENV-1,-3, and-4 (44). The tetravalent TAK-003 formulation was also shown to be safe, immunogenic, and protective against lethal DENV challenge in both rodent models and non-human primates (37, 44, 45). In clinical trials, TAK-003 is well-tolerated and capable of generating significant humoral immunity against all four DENV serotypes in both children and adults, regardless of previous dengue serostatus (46–51). Previous analysis of the T cell cytokine production profile generated by TAK-003 administration in flavivirus-naïve recipients demonstrated that this product generates a pool of NS1, NS3, and NS5 reactive CD8^+^ T cells capable of producing IFN-γ, TNF-α, and to a lesser extent IL-2 upon *ex vivo* restimulation (52). However, the magnitude, stability, and antigen specificity of cell-mediated immunity generated in response to a TAK-003 vaccination has not previously been described in detail.

In this study, we demonstrate that a single dose of TAK-003 elicits a potent T cell response as assessed by IFN-γ ELISPOT 28- and 120-days post vaccination. Reactivity against the structural genes of DENV-1,-2,-3, and-4 contained within the vaccine formulation was observed to be significantly elevated over pre-vaccination levels 28 days post vaccination, with reactivity against the structural proteins of DENV-2 and-4 maintained for at least 120 days. However, reactivity against the structural regions of DENV represented only 13–20% of the total T cell response observed, with the rest of the T cell response directed against the non-structural DENV proteins. As the PDK-53-derived DENV-2 backbone is the only source of non-structural DENV antigen in TAK-003, a significant amount of reactivity against these gene products is observed, with reactivity against DENV-2 NS1, NS3, and NS5 dominating. While there are no DENV-1,-3, and-4 non-structural antigens present in TAK-003, a significant amount of T cell cross-reactivity against these antigens was observed 28- and 120-days post vaccination. The magnitude of cross-reactivity observed was directly proportional to the strength of the response directed against the DENV-2 non-structural antigens contained within TAK-003. While the overall response rate to vaccination as measured by IFN-γ ELISPOT was very high in this study (85% at day 120), significant individual-to-individual variability was observed in antigen immunodominance hierarchy. Dimensional-reduction visualization (tSNE projection) and hierarchal cluster analysis of ELISPOT reactivity data revealed the presence of 7 distinct patterns of DENV-2 reactivity following TAK-003 vaccination. Individuals falling within these distinct clusters of reactivity maintained their relative cluster localization between 28- and 120-days post vaccination, suggesting that these designations represent stable outcomes of vaccination. Finally, detailed epitope mapping of T cell reactivity against the DENV-2 proteome using a matrix of overlapping peptide pools demonstrated that TAK-003 elicits a broad response directed across the DENV-2 proteome.

## Materials and Methods

### Cells/Samples

The samples used in this study were collected during a Phase 1 trial in US adults of a tetravalent, live-attenuated dengue virus vaccine candidate, TAK-003 (“Impact of Subcutaneous vs. Intramuscular Administration of Inviragen's Live Attenuated Dengue Vaccine on Safety and Immunogenicity” NCT01728792; WRAIR #1987). Subjects received one dose of TAK-003 either subcutaneously or intramuscularly and whole blood was collected at day of vaccination (day 0) and at days 14, 28, and 120 post-vaccination. Individuals were not stratified by route of vaccination in subsequent analysis. Whole blood was collected in Cell Preparation Tubes (BD Vacutainer) for isolation of PBMC. Cells were cryopreserved at ~10^7^ per mL and stored in vapor-phase liquid nitrogen until use. Vaccine administration and PBMC collection were performed after written informed consent in accordance with the Declaration of Helsinki. The studies and protocols were approved by the institutional review boards at the State University of New York Upstate Medical University and the Human Subjects Research Review Board for the Commanding General of the U.S. Army Medical Research and Material Command. Exclusion criteria for participation in this study include history of dengue fever, Japanese Encephalitis, West Nile or Yellow Fever disease, and history of travel to dengue endemic areas including the Caribbean, Mexico, Central America, South America or Southeast Asia during the month prior to screening, or planned travel to a dengue endemic area during the study period. All subjects were screened to be seronegative to dengue or West Nile at the time of study initiation.

### T Cell ELISPOT Assay

Cryopreserved PBMC were thawed and placed in RPMI 1640 medium supplemented with 10% heat-inactivated normal human serum (100–318, Gemini Bio-Products), L-glutamine, penicillin, and streptomycin. After an overnight rest at 37°C, the PBMC were washed, resuspended in serum free medium (SFM; X-VIVO 15, Lonza), cellular viability assessed by trypan blue exclusion, and 1–2 × 10^5^ viable cells were plated per well of a 96-well Millipore MAIPSWU plate coated with anti-IFN-γ antibody according to the manufacturer's instructions (3420-2HW-Plus, Mabtech Inc.). Peptide pools were added to the cells at a final concentration of 1 μg/mL/peptide prior to incubation at 37°C overnight. Controls included SFM plus 0.5% DMSO (negative) and anti-CD3 (positive). The ELISPOT plates were developed using TMB substrate and read using a CTL-ImmunoSpot® S6 Ultimate-V Analyzer (Cellular Technology Limited). All peptide pools were tested in duplicate, and the mean value of the duplicate wells utilized as the reported value. Individuals were considered reactive to a peptide pool when the response was >50 SFC/10^6^ PBMC and was 4-fold over the corresponding negative control. For matrix ELISPOT analysis, positive wells were defined as those with a signal 5-fold over the negative (no stimulation) control, and >5 spots per well after subtracting the negative (no stimulation) control. All data were normalized based on the number of cells plated per well and are presented herein as SFC/10^6^ PBMC values.

### Peptides

Overlapping peptide pools corresponding to the full-length envelop (E), non-structural 1 (NS1), NS3, and NS5 proteins for DENV-1-4 and NS2a/b and NS4a/b proteins for DENV-2 were obtained through the NIH Biodefense and Emerging Infections Research Resources Repository, NIAID, NIH (Supplemental Table 4). Additional overlapping peptide pools covering the capsid (C) and precursor membrane (prM) proteins of DENV-1-4, were purchased from JPT Peptide Technologies (Supplemental Table 4). Peptide pool stocks were reconstituted in DMSO at a concentration of 200 μg/mL/peptide and stored at −80°C.

### Statistical Analysis

TSNE projection, hierarchical clustering, and visualization of multidimensional IFN-γ ELISPOT data was performed in R (v3.5.2). tSNE projection and hierarchical clustering was calculated using the package rtsne utilizing background-subtracted data, a perplexity value of 30 and theta value of 0.5. Hierarchical clustering was performed using a cluster value of 7. The resulting two-dimensional dataset was visualized using the package ggplot2. All code is available upon request from the corresponding author. All other statistical analyses were performed using GraphPad Prism v6 Software (GraphPad Software, La Jolla, CA). A *p*-value <0.05 was considered significant.

## Results

### TAK-003 Elicits Significant Cellular Immunity Against the Structural Proteins of DENV-1,-2,-3, and -4

**Figure 1:**
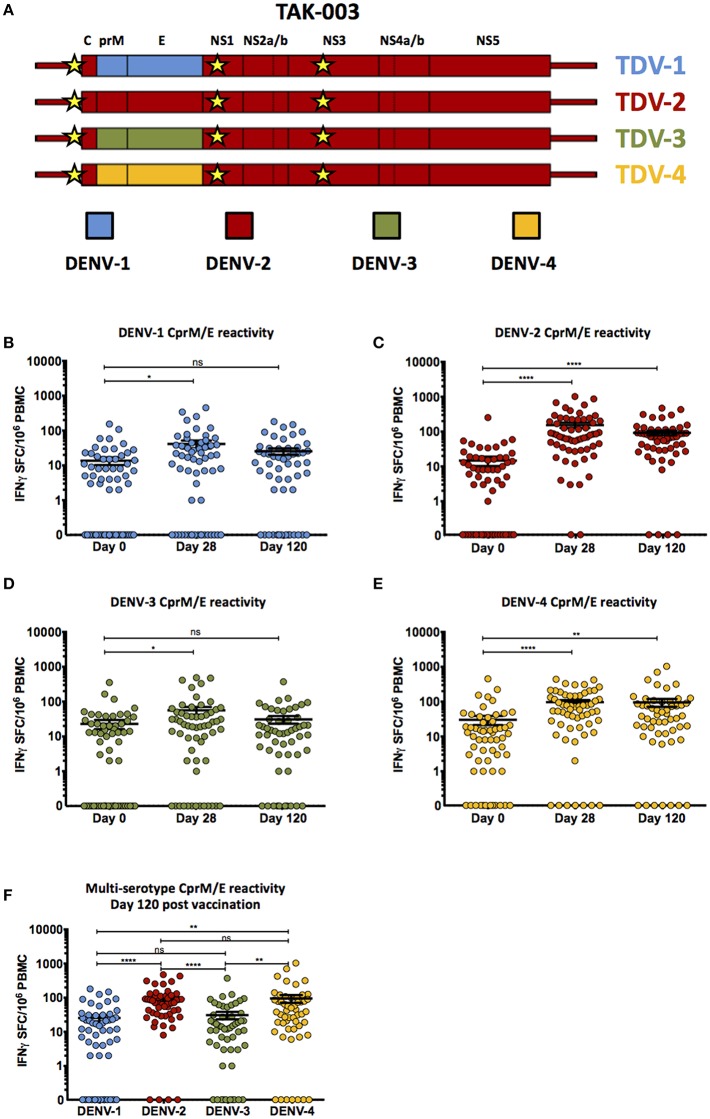
Cell-mediated reactivity against DENV-1,-2,-3, and-4 structural proteins following TAK-003 administration. **(A)** Schematic representation of the genetic structure of the tetravalent TAK-003 vaccine platform. Key attenuating mutations in the 5′ UTR, NS1, and NS3 are indicated by the yellow stars. **(B)** Frequency of IFN-γ producing cells as quantified by ELISPOT following DENV-1 CprM/E peptide stimulation at the indicated time points following vaccination. **(C)** Frequency of IFN-γ producing cells as quantified by ELISPOT following DENV-2 CprM/E peptide stimulation at the indicated time points following vaccination. **(D)** Frequency of IFN-γ producing cells as quantified by ELISPOT following DENV-3 CprM/E peptide stimulation at the indicated time points following vaccination. **(E)** Frequency of IFN-γ producing cells as quantified by ELISPOT following DENV-4 CprM/E peptide stimulation at the indicated time points following vaccination. **(F)** Frequency of IFN-γ producing cells as quantified by ELISPOT following DENV-1,-2,-3, and-4 CprM/E peptide stimulation at day 120 post vaccination. *n* = 60 for days 0 and 28, *n* = 54 for day 120. ^*^p < 0.05, ^**^p < 0.01, ^****^p < 0.0001, ns, not significant Paired two-tailed *t*-test. Bars indicate group mean ± SEM. Zero values were superimposed on the logarithmic graph.

TAK-003 is a recombinant, tetravalent, live-attenuated dengue vaccine candidate derived from the attenuated PDK-53 DENV-2 strain. To generate a vaccine product which contains the structural proteins from all four DENV serotypes, the structural genes (CprM/E) from the parental DENV-2 backbone were sequentially replaced with those from DENV-1, DENV-3, or DENV-4 to create three additional recombinant DENV strains (Figure 1A). These four live-attenuated viruses were co-formulated for simultaneous vaccine administration.

Vaccination with TAK-003 resulted in a significant increase in the number of circulating IFN-γ producing cells at day 28 post vaccination responding to the structural proteins (CprM/E) from DENV 1 (Figure 1B, Table 1), DENV-2 (Supplemental Figure 1, Figure 1C, Table 1), DENV-3 (Figure 1D, Table 1), and DENV-4 (Figure 1E, Table 1) as assessed by IFN-γ ELISPOT. However, while the number of circulating IFN-γ producing cells reacting to the structural proteins (CprM/E) of DENV-2 and DENV-4 remained significantly elevated on day 120 post vaccination relative to pre-vaccination (Figures 1C,E), the number of circulating DENV-1 CprM/E and DENV-3 CprM/E reactive cells on day 120 post vaccination was returning to baseline (pre-vaccination) levels when assessed as a group (Figures 1B,D). Therefore, despite significant reactivity against CprM/E derived from all serotypes at 28-days post vaccination, there were significantly more DENV-2 and DENV-4 reactive T cells on day 120 than DENV-1 and DENV-3 reactive cells (Figure 1F).

**Table 1:** Percent of subjects reactive to DENV structural proteins.

	**Percent of subjects reactive to DENV structural proteins**								
		**C/prM**				***E***			
	***N***	**DENV-1**	**DENV-2**	**DENV-3**	**DENV-4**	**DENV-1**	**DENV-2**	**DENV-3**	**DENV-4**
Day 0 (Pre-vaccination)	60	0%	0.0%	0%	5%	0%	0.0%	1.7%	1.7%
Day 28 (Post-vaccination)	54	1.7%	16.7%	1.7%	16.7%	6.7%	26.7%	6.7%	6.7%
Day 120 (Post-vaccination)	54	0%	9.3%	1.9%	18.5%	3.7%	22.2%	3.7%	9.3%

### TAK-003 Elicits Significant and Broad Cellular Immunity Against the Non-structural Proteins of DENV-2

While the majority of the protective humoral immunity generated by DENV infection or vaccination is canonically thought to be directed against the DENV envelope protein (53–56), a significant fraction of the cell-mediated immune response to DENV infection is directed against the non-structural proteins (NS1, NS2a/b, NS3, NS4a/b, and NS5) (33, 34). As the only non-structural DENV proteins contained within the TAK-003 formulation originate from the DENV-2 backbone (Figure 1A), we assessed the magnitude and breadth of the T cell response generated against all five DENV-2 NS proteins on days 28 and 120 post vaccination.

**Figure 2:**
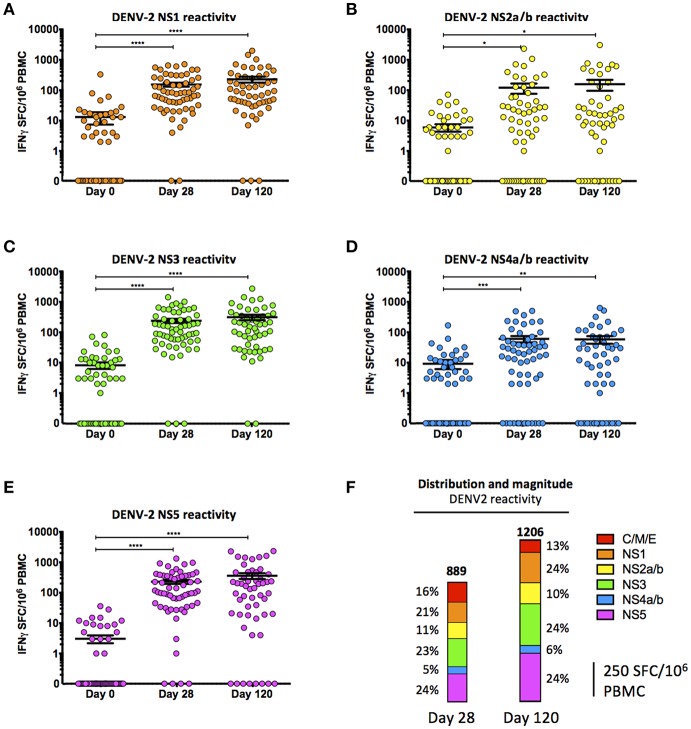
Cell-mediated reactivity against DENV2 non-structural proteins following TAK-003 administration. **(A)** Frequency of IFN-γ producing cells as quantified by ELISPOT following DENV-2 NS1 peptide stimulation at the indicated time points following vaccination. **(B)** Frequency of IFN-γ producing cells as quantified by ELISPOT following DENV-2 NS2a/b peptide stimulation at the indicated time points following vaccination. **(C)** Frequency of IFN-γ producing cells as quantified by ELISPOT following DENV-2 NS3 peptide stimulation at the indicated time points following vaccination. **(D)** Frequency of IFN-γ producing cells as quantified by ELISPOT following DENV-2 NS4a/b peptide stimulation at the indicated time points following vaccination. **(E)** Frequency of IFN-γ producing cells as quantified by ELISPOT following DENV-2 NS5 peptide stimulation at the indicated time points following vaccination. **(F)** Relative distribution and magnitude of DENV2 reactivity at day 28 and 120 post vaccination. Numbers at the top of each bar indicate average number of Spot Forming Cells (SFCs) per million PBMCs observed in response to DENV-2 stimulation in all subjects at the relevant time points. *n* = 60 for days 0 and 28, *n* = 54 for day 120. ^*^p < 0.05, ^**^p < 0.01, ^***^p < 0.001, ^****^p < 0.0001 Paired two-tailed *t*-test. Bars indicate group mean ± SEM. Zero values were superimposed on the logarithmic graph.

Immunization with TAK-003 resulted in a significant increase in the number of circulating IFN-γ producing T cells at day 28 post vaccination responding to DENV-2 NS1 (Supplemental Figure 1, Figure 2A, Table 2), DENV-2 NS2a/b (Supplemental Figure 1, Figure 2B, Table 2), DENV-2 NS3 (Supplemental Figure 1, Figure 2C, Table 2), DENV-2 NS4a/b (Supplemental Figure 1, Figure 2D, Table 2), and DENV-2 NS5 (Supplemental Figure 1, Figure 2E, Table 2). These responses persisted for at least 120 days post vaccination, some of which increased moderately from day 28 to day 120 (Figures 2A–E). Responses to non-structural proteins accounted for ~85% of the total reactivity against DENV-2, with NS1, NS3, and NS5 contributing a combined ~75% of the overall DENV-2 response (Figure 2F). While the total number of IFN-γ producing DENV-2 reactive T cells increased from day 28 to day 120 post vaccination, the relative distribution of the responses to individual DENV-2 structural and non-structural proteins remained consistent.

**Table 2:** Percent of subjects reactive to the DENV-2 proteome.

	**Percent of subjects DENV-2 proteome reactive**								
	***N***	**Total proteome**	**C/prM**	***E***	**NS1**	**NS2a/b**	**NS3**	**NS4a/b**	**NS5**
Day 0 (Pre-vaccination)	60	1.7%	0.0%	0.0%	1.7%	0.0%	0.0%	0.0%	0.0%
Day 28 (Post-vaccination)	54	85.0%	16.7%	26.7%	41.7%	20.0%	51.7%	16.7%	53.3%
Day 120 (Post-vaccination)	54	81.5%	9.3%	22.2%	42.6%	18.5%	61.1%	20.4%	55.6%

### TAK-003 Elicits DENV Serotype Cross-Reactive Responses Against the Non-structural Proteins of DENV-1,-3, and -4

**Figure 3:**
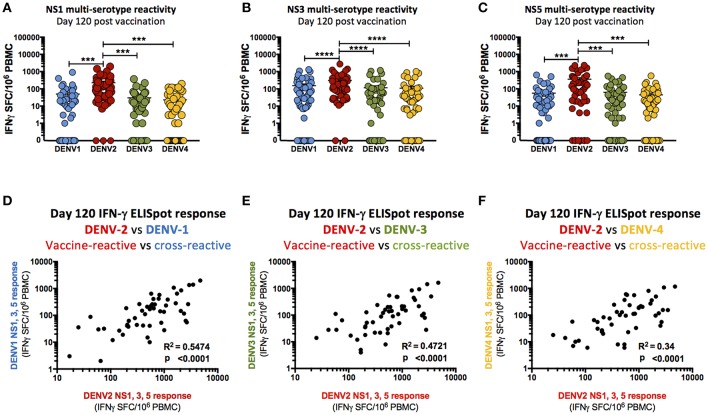
Cross-reactivity of T cell responses following TAK-003 administration. **(A)** Frequency of IFN-γ producing cells as quantified by ELISPOT upon DENV-1,-2,-3, and-4 NS1 peptide stimulation at day 120 post vaccination. **(B)** Frequency of IFN-γ producing cells as quantified by ELISPOT upon DENV-1,-2,-3, and-4 NS3 peptide stimulation at day 120 post vaccination. **(C)** Frequency of IFN-γ producing cells as quantified by ELISPOT upon DENV-1,-2,-3, and-4 NS5 peptide stimulation at day 120 post vaccination. **(D)** Relative frequency of IFN-γ producing cells activated in response to DENV-1 NS1, NS3, and NS5 vs. DENV-2 NS1, NS3, and NS5 peptide stimulation at day 120 post vaccination. **(E)** Relative frequency of IFN-γ producing cells activated in response to DENV-3 NS1, NS3, and NS5 vs. DENV-2 NS1, NS3, and NS5 peptide stimulation at day 120 post vaccination. **(F)** Relative frequency of IFN-γ producing cells activated in response to DENV-4 NS1, NS3, and NS5 vs. DENV-2 NS1, NS3, and NS5 peptide stimulation at day 120 post vaccination. *n* = 54. ^***^p < 0.001, ^****^p < 0.0001 Paired two-tailed *t*-test. R^2^ calculated by linear regression. Bars indicate group mean ± SEM. Zero values were superimposed on the logarithmic graph.

While the only non-structural DENV genes contained within TAK-003 are DENV-2 in origin, a significant cellular immune response to the non-structural proteins (NS1, NS3, NS5) of DENV-1,-3, and-4 was observed 28- and 120-days post TAK-003 administration as assessed by IFN-γ ELISPOT (Supplemental Figure 2, Table 3). The only exception to this trend was the cross-reactive response observed against DENV-1 NS1. While some individuals did have a detectible DENV-1 NS1 response (Table 3) the overall magnitude of all subjects failed to reach significance at either 28- or 120-days post vaccination (Supplemental Figure 1). While the overall magnitude of the observed cross-reactive response against most non-structural proteins of DENV-1,-3, and-4 was significantly elevated relative to baseline following TAK-003 administration, the relative responses to NS1 (Figure 3A), NS3 (Figure 3B), and NS5 (Figure 3C) from DENV-1,-3, and-4 were significantly lower than the responses observed against the corresponding proteins from DENV-2. These data suggest that any reactivity against the non-structural proteins of DENV-1, -3, and-4 is dependent on a strong response against the non-structural proteins of DENV-2. This hypothesis is supported by the observation that the magnitude of TAK-003-stimulated DENV-1 (Figure 3D), DENV-3 (Figure 3E) and DENV-4 (Figure 3F) non-structural protein cross-reactivity is directly proportional to the magnitude of the corresponding DENV-2 non-structural protein response. Therefore, while multi-serotype cellular immunity against DENV non-structural proteins can be generated in response to what is essentially a monovalent exposure, the magnitude of cross-reactivity is significantly lower and directly proportional to the magnitude of the vaccine-directed response.

**Table 3:** Percent of subjects reactive to DENV non-structural proteins.

	**Percent of subjects reactive to DENV non-structural proteins**												
		**NS1**				**NS3**				**NS5**			
	***N***	**DENV-1**	**DENV-2**	**DENV-3**	**DENV-4**	**DENV-1**	**DENV-2**	**DENV-3**	**DENV-4**	**DENV-1**	**DENV-2**	**DENV-3**	**DENV-4**
Day 0 (Pre-vaccination)	60	3.3%	1.7%	0%	1.7%	1.7%	0.0%	0%	0%	1.7%	0.0%	0%	3.3%
Day 28 (Post-vaccination)	54	8.5%	41.7%	10.2%	6.8%	26.7%	51.7%	26.7%	21.7%	15%	53.3%	18.3%	11.7%
Day 120 (Post-vaccination)	54	5.6%	42.6%	9.3%	6.7%	37%	61.1%	25.9%	22.2%	20.4%	55.6%	20.4%	13%

### Distinct Patterns of Antigen Reactivity Following TAK-003 Administration

Although administration of TAK-003 resulted in a significant and fairly uniform overall increase in total DENV-2 reactivity 28- and 120-days post vaccination (Table 2), there was significant individual-to-individual variation in the pattern of DENV-2 protein reactivity. As has been observed following natural DENV infection or vaccination with other DENV vaccine candidates, individuals often exhibit differences in antigen immunodominance hierarchy (33, 34). This variation in individual DENV epitope reactivity can be partially attributed to differences in host HLA genotype, which dictates which DENV-derived epitopes can be efficiently presented by infected cells or professional antigen presenting cells to putative DENV-reactive T cells (28).

In an attempt to capture and quantify the unique individual patterns of DENV reactivity elicited by TAK-003 administration in an unsupervised fashion—and to potentially define statistically unique patterns of reactivity that might eventually correlate with vaccine efficacy or durability—we utilized dimensional compression analysis to display the magnitude and specificity of the IFN-γ ELISPOT response directed against the DENV-2 proteome (CprM, E, NS1, NS2a/b, NS3, NS4a/b, and NS5) on days 0, 28, and 120 post vaccination in 2-dimensional space. We used t-Distributed Stochastic Neighbor Embedding (tSNE) visualization and hierarchical cluster analysis to visualize and group 174 data points generated during this study. Each data point corresponds to one time point for one subject, and reflects the cumulative reactivity against both the structural and non-structural proteins of DENV-2.

**Figure 4:**
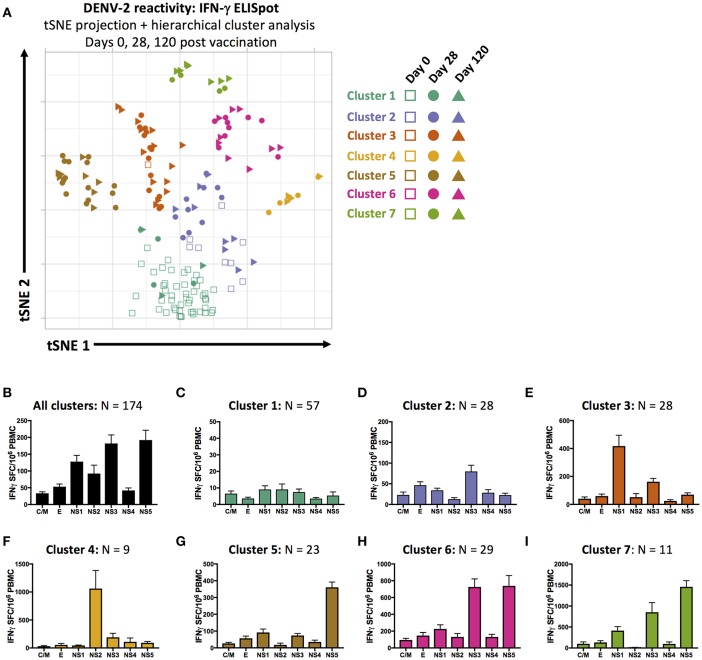
Unsupervised dimensional reduction and hierarchal clustering of DENV-2 antigen reactivity. **(A)** tSNE projection and hierarchal clustering of 7-dimentional DENV-2 reactivity data (C/M, E, NS1, NS2a/b, NS3, NS4a/b, and NS5 stimulation) as assessed by IFN-γ ELISPOT in all samples from days 0, 28, and 120 post TAK-003 administration. Average frequency of IFN-γ producing cells as assessed by ELISPOT in response to stimulation with the indicated peptide pools in **(B)** all data points, **(C)** cluster 1 data points, **(D)** cluster 2 data points, **(E)** cluster 3 data points, **(F)** cluster 4 data points, **(G)** cluster 5 data points, **(H)** cluster 6 data points, or **(I)** cluster 7 data points. Bars indicate group mean ± SEM. tSNE clustering was performed with a perplexity value of 30 and theta of 0.5. Hierarchical clustering analysis was performed using a cluster value of 7.

Using these tools, we were able to identity 7 statistically distinct clusters of DENV-2 reactivity in TAK-003 recipients on days 0, 28, or 120 post vaccination (Figures 4A,B). Data points falling in cluster 1 possess little-or-no DENV reactivity, and overwhelmingly correspond to pre-vaccination samples (Figure 4C). Cluster 2 contains a mix of day 0, 28, and 120 post vaccination samples. While exhibiting slightly higher DENV-2 reactivity than cluster 1, cluster 2 does not exhibit a distinct pattern of reactivity and contains those individuals who responded poorly to vaccination or whose response to vaccination did not persist (Figure 4D). Data points falling in clusters 3 and 4 are from primarily day 28 and day 120 post vaccination samples and exhibit a strong NS1 or NS2a/b biased response, respectively (Figures 4E,F). Cluster 5 is characterized by a dominant NS5 response (Figure 4G), while clusters 6 and 7 have dual NS3 and NS5 reactivity of differing magnitudes (Figures 4H,I).

Since our analysis contained multiple time points for each individual post vaccination, we were able to assess the stability of our statistically defined clusters as TAK-003-elicted immunity matured over time, and how localization in a given cluster corresponded to an individual's long-term DENV reactivity. Only two individuals fell within cluster 1 at day 28 post vaccination and either remained in cluster 1 on day 120 post vaccination or moved to the poorly-responsive cluster 2 (Supplemental Figure 3, Supplemental Table 1), suggesting that these individuals are true non-responders to vaccination from the perspective of T cell immunity. As predicted by the low-level and relatively non-specific DENV-2 reactivity that characterizes cluster 2, this cluster is highly unstable between days 28 and 120 post vaccination and likely represents a “transition” state between more stable outcomes of vaccination (Supplemental Figure 3, Supplemental Table 1). Individuals falling within cluster 3 and cluster 4 on day 28 post vaccination (NS1 or NS2a/b reactive) are highly stable, with 82% of individuals staying within the same cluster between days 28 and 120 post vaccination (Supplemental Figure 3, Supplemental Table 1). There is significant movement of individuals between clusters 5, 6 and 7 between days 28 and 120, but little movement of individuals out of this “super-group.” As clusters 5, 6, and 7 are characterized by different magnitudes of reactivity against the same antigens (NS3 and/or NS5), movement between these clusters might be predicted as immunity stabilizes after vaccination. No information is currently available on how these clusters may correspond to protection against subsequent challenge or infection, but these data demonstrate a novel method to assess the diversity and stability of vaccine-elicited cellular immunity.

### Epitope Mapping of DENV-2 Reactivity Day-120 Post Vaccination

In light of the complex—yet restricted—pattern of DENV-2 reactivity observed following TAK-003 administration, we endeavored to define the exact DENV-2 derived epitopes recognized by the immune system following vaccination. To this end, we performed a matrix ELISPOT analysis of PBMC samples from 40 individuals obtained 120 days post TAK-003 vaccination using a library of 532 overlapping peptides spanning the entire DENV-2 proteome. The peptides used were 15-20 AA in length, overlapping by 10–14 AA, and were derived from the DENV-2 NGC strain.

**Figure 5:**
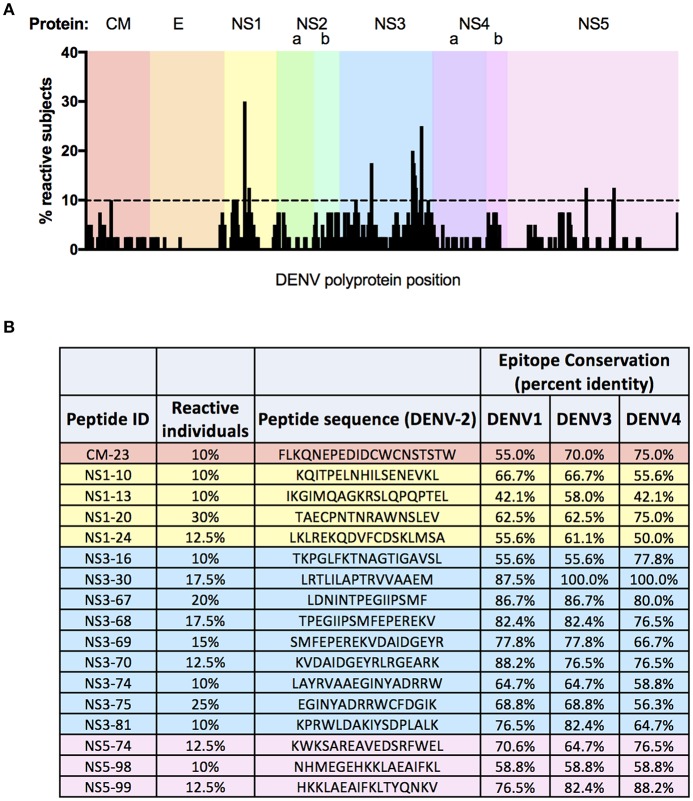
Fine epitope mapping of DENV-2 T cell responses following TAK-003 administration. Magnitude and distribution of DENV-2 reactivity in TAK-003 recipients 120 days post vaccination. **(A)** Schematic representation of DENV-2 epitope reactivity following TAK-003 vaccination across the DENV-2 proteome. Bar height indicates the percentage of subjects responding to the indicated peptide at the corresponding genomic location. **(B)** Immunodominant DENV-2 epitopes identified in this analysis, defined as peptides eliciting a response in 10% or more of study participants following vaccination. Conservation of identified immunodominant DENV-2 peptides shown relative to DENV-1 (Singapore/S275/1990), DENV-3 (Philippines/H87/1956), and DENV-4 (Singapore/8976/1995). Conservation calculated as percent identify at the amino acid level. *n* = 40 subjects.

A total of 221 peptides from the library of 532 possible candidates were recognized as reactive across all 40 individuals screened in this assay (Figure 5A, Supplemental Table 2). Of these immunogenic peptides, 109 were recognized by 5% or more of subjects analyzed (Supplemental Table 3), while 17 peptides were recognized by more than 10% of subjects (Figure 5B). The frequency of observed reactivity was highest for peptides derived from NS3, with 79.5% of all peptides derived from this protein eliciting a positive response in at least one subject, and with each peptide being recognized by 4.7% of subjects on average (Supplemental Table 2). In contrast, only 13.4% of peptides derived from E were recognized as immunogenic in TAK-003 immunized individuals, with each peptide being recognized by 0.48% of subjects on average. While proportionally the majority of the immunogenic peptides identified in this analysis were derived from NS3 (18.5% of all positive peptides), the most commonly recognized peptide screened in this analysis was derived from NS1, with 30% of subjects exhibiting reactivity against this single peptide. Notably, 90% of subjects falling into the previously described NS1-reactive “cluster 3” (Figure 4A) exhibited reactivity against this single dominant NS1-derived peptide or its adjacent neighbor (with which it shares 10 of 16 AA), suggesting that responsiveness to this epitope may be a significant driving factor in determining NS1- biased reactivity following vaccination.

On average, amino acid level sequence conservation (identity) is 70% between all DENV serotypes (57). However, there is variation in the extent of sequence conservation in the various DENV protein subunits, including regions compatible with HLA presentation (33, 34). This fact is reflected in the relative amino acid level conservation of the 17 dominant immunogenic peptides identified in our screen. For those peptides derived from DENV-2 NS1 identified as immunogenic in this analysis, the average amino-acid level conservation (identity) between DENV-2 and the other 3 DENV serotypes is only 58%. In contrast, the immunogenic peptides derived from DENV-2 NS3 shared on-average 76% amino-acid level identity with NS3 from the other 3 DENV serotypes. These values offer a potential mechanistic explanation for the weak DENV-1 NS1 cross-reactivity observed in TAK-003 recipients, as well as the correspondingly robust NS3 cross-reactivity (Supplemental Figure 2).

## Discussion

In this study, we demonstrate that a single dose of the candidate tetravalent DENV vaccine TAK-003 elicits DENV-specific T cell responses in humans as assessed by IFN-γ ELISPOT at both 28- and 120-days post immunization. These responses were directed against all the antigens contained within the formulation, including the structural regions of DENV-1 to-4, as well as the non-structural regions of the DENV-2 derived vaccine backbone. In addition, significant cross-reactivity against the non-structural gene products (NS1, NS3, and NS5) of DENV-1,-3, and-4 was observed, with the magnitude of the cross-reactivity dictated by the magnitude of the response generated by the non-structural gene products of the vaccine's DENV-2 backbone. While the fraction of individuals responding to TAK-003 vaccination as assessed by IFN-γ ELISPOT was very high (85% at day 28), significant individual-to-individual variation in the pattern of antigenic reactivity was observed. We were able to categorize individuals into statistically distinct clusters based on their pattern of antigen reactivity using unsupervised dimensional reduction projection/visualization and hierarchical clustering of the IFN-γ ELISPOT data. Finally, we performed fine-epitope mapping of the DENV-2 T cell responses generated by TAK-003 vaccination using a matrix of 532 overlapping peptides representing the entire DENV-2 proteome. This approach identified 221 potential T cell epitopes spread across the DENV-2 proteome, of which 109 were found to be immunogenic in 5% or more study-subjects following vaccination, while 17 peptides were found to be immunogenic in 10% or more study-subjects.

Assessing and leveraging cell-mediated immunity in the setting of dengue vaccine design is increasing in relevance and urgency in light of the weak correlation observed between NAb titers and protective immunity following vaccination with Dengvaxia®) (15–17). While neutralizing antibodies are necessary and sufficient to provide sterile immunity to viral infection in animal models (58), growing evidence suggests that they are not sufficient to provide complete protection from infection in humans (15–17). Vaccine-directed cell-mediated immunity can in theory restrict viral replication and dissemination following DENV infection without risking the development of severe dengue via antibody-mediated immune enhancement during waning immunity. Furthermore, the development of stable, diverse, and mature humoral immunity is dependent on T cell help during B cell germinal center maturation (59). All vaccine products can reasonably be expected to induce some form of cell-mediated immunity—whether it is direct effector cells such as classical CTL, or NK cells, helper T cells which modulate the antibody and CTL responses, or more likely some combination of these. However, ensuring and confirming that the antigen-specific cellular immune response (T cell mediated) is directed against antigens that can enable lysis of infected cells or facilitate maturation of nascently activated B cells has not always been a focus in immunogenicity studies of candidate dengue vaccines.

In addition to producing durable and functional immunity, a key consideration for DENV vaccine design is generating immunity against four immunologically distinct viruses in a balanced and simultaneous manner. There is significant amino acid level homology between the four DENV serotypes (~70%), meaning that immunological cross-reactivity is readily achieved for both humoral and cell-mediated immunity, albeit with varying degrees of balance. However, the role that cross-reactive T cells play in facilitating DENV immunity and/or pathology is complex and incompletely understood. Severe dengue is often associated with significant peripheral T cell activation and expansion, including a significant number of serotype cross-reactive T cells (35, 60–62). Prior DENV exposure appears to have little impact on the number and frequency of DENV cross-reactive cells activated in response to acute infection, although the timing of their expansion and circulation differs between primary and secondary infection. However, as clinical symptoms of severe dengue (plasma leakage, etc) occur with or after defervescence and viral clearance, severe dengue is a consequence of dysregulated immunity following symptomatic viral infection (63). Therefore, categorizing cross-reactive T cells as either pathogenic or protective based on their presence during severe dengue is potentially problematic, and may misconstrue cause-and-effect. As there are currently no data available on the relationship between the generation of cross-reactive T cells following vaccination and protection from infection or severe disease, the precise role these cells play in protective immunity—especially in previously flavivirus-naïve individuals—remains unclear.

In addition to quantifying the cellular immunogenicity of the candidate DENV vaccine TAK-003, this study highlights two important features of DENV cellular immunity which can help refine approaches for functionally assessing future vaccines. Firstly, caution should be exercised when down-selecting epitopes or antigens to assess cellular immunity following vaccination or natural exposure to DENV. While NS3-derived peptides dominate the list of immunogenic epitopes identified in this analysis and NS3 responses exhibit the most cross-reactivity (presumably due to NS3 sequence conservation between serotypes), a significant fraction of vaccinated individuals in this trial exhibited little-or-no reactivity against NS3, and instead exhibited monotypic reactivity against NS1, NS2a/b, or NS4a/b. These distinct and stable patterns of individual reactivity are most likely attributable to differences in host HLA genotype. Whether or not these unique patterns of reactivity correlate with protection following vaccination is still unclear, but the observation underlines the prudence of assessing the immunogenicity of a broad panel of vaccine-derived antigens. Secondly, due to the unique design of the TAK-003 vaccine product, this study is among the first to directly assess the magnitude and persistence of vaccine-elicited DENV serotype-cross-reactivity in previously flavivirus-naïve individuals. We observed that the magnitude of serotype cross-reactivity for any given antigen is directly proportional to the magnitude of the response elicited by the corresponding vaccine-component. This observation provides mechanistic insight into the pattern of vaccine-driven immunity, and can also guide future vaccine design. Notably, while reactivity against the structural regions of DENV-1 and DENV-3 (antigens contained within the vaccine formulation) wanes by 120-days post vaccination, significant reactivity against the non-structural regions of DENV-1,-3, and-4 can still be observed at 120-days, despite the fact that these antigens are not contained within the vaccine formulation. The persistence of this cross-reactive response is attributable to the fact that the non-structural regions of the TAK-003 DENV-2 backbone accounts for the majority of the cellular immune response observed following vaccination. While the cross-reactive immune response directed against the non-structural regions of DENV-1,-3, and-4 is significantly lower than the vaccine-elicited response to the same regions of DENV-2, the relative magnitude of these responses still means that cross-reactive cellular immunity can persist even when responsiveness to other vaccine components wanes.

## Ethics Statement

The investigators have adhered to the policies for protection of human subjects as prescribed in AR 70–25. The studies were approved by the institutional review boards at the State University of New York Upstate Medical University and the Human Subjects Research Review Board for the Commanding General of the U.S. Army Medical Research and Material Command.

## Author Contributions

MG and AK generated the data. JC conceived of the analysis, designed the experiments, and analyzed the data. RJ provided project oversite and secured funding. TE and ST conceived and designed the clinical trial. MP executed and oversaw the clinical trial and collected samples. HF designed and executed experiments and analyzed the data. AW analyzed the data and wrote the manuscript.

### Conflict of Interest Statement

The authors declare that the research was conducted in the absence of any commercial or financial relationships that could be construed as a potential conflict of interest.
