# Supplementary material for: Assessing the Diversity and Stability of Cellular Immunity Generated in Response to the Candidate Live-Attenuated Dengue Virus Vaccine TAK-003
Source: Front Immunol. 2019 Jul 31;10:1778. doi: 10.3389/fimmu.2019.01778 (PMC6684763; doi:10.3389/fimmu.2019.01778)
Supplement: Supplementary file 1 [file Presentation_1.PPTX]

## Slide 1
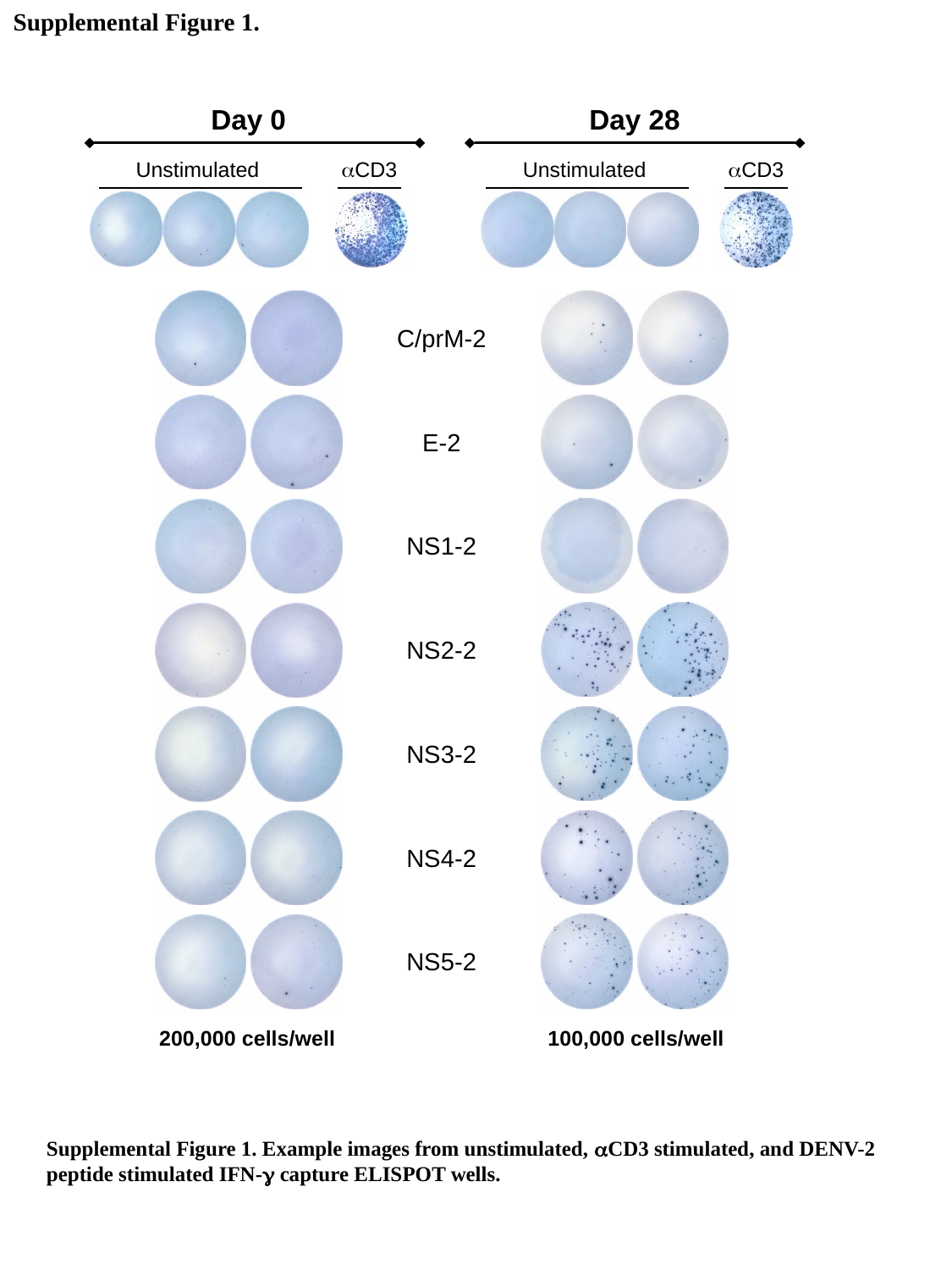

Supplemental Figure 1.
Day 0
Day 28
Unstimulated
aCD3
Unstimulated
aCD3
C/prM-2
E-2
NS1-2
NS2-2
NS3-2
NS4-2
NS5-2
100,000 cells/well
200,000 cells/well
Supplemental Figure 1. Example images from unstimulated, aCD3 stimulated, and DENV-2 peptide stimulated IFN-g capture ELISPOT wells.

## Slide 2
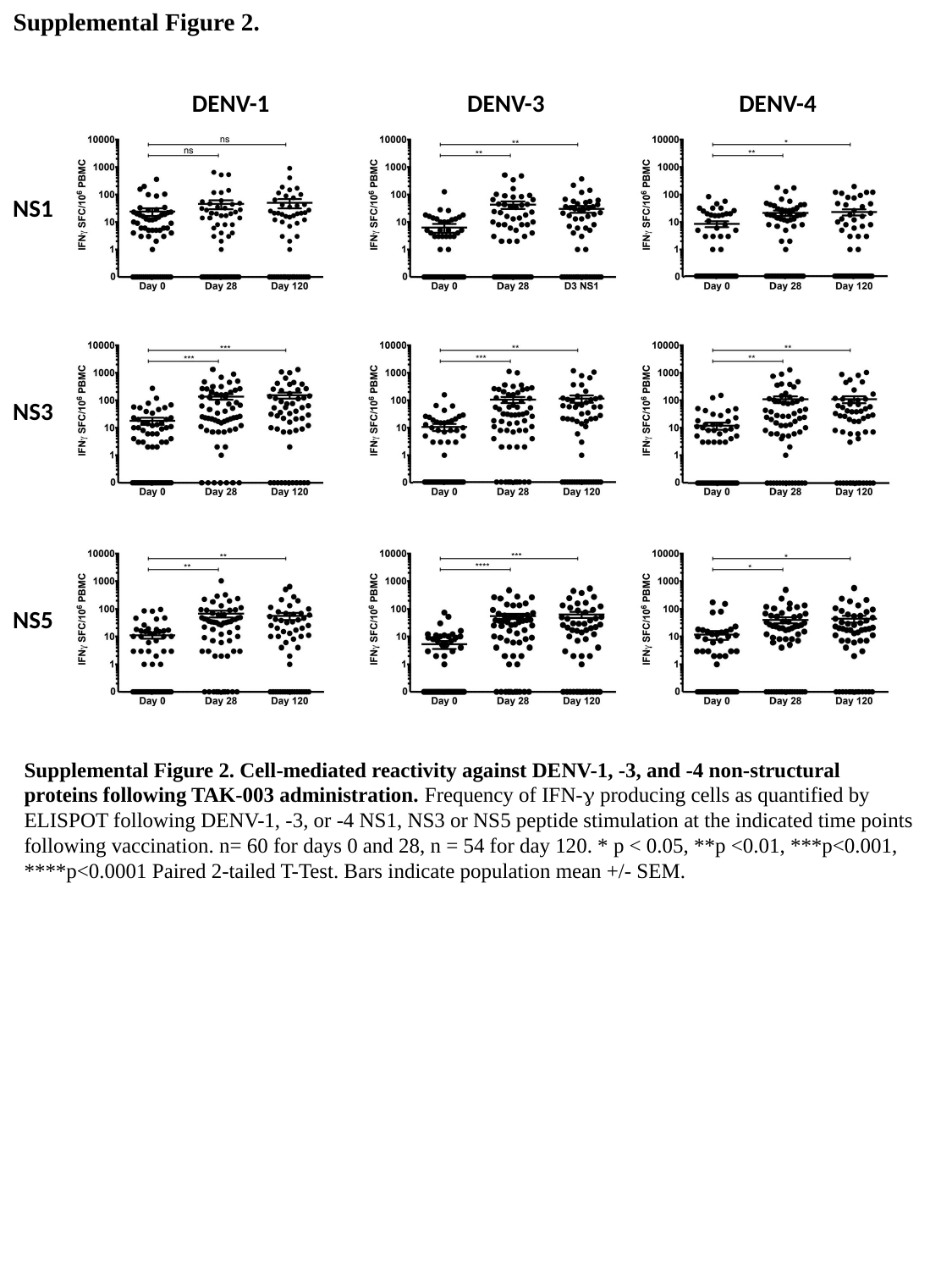

Supplemental Figure 2.
DENV-1
DENV-3
DENV-4
NS1
NS3
NS5
Supplemental Figure 2. Cell-mediated reactivity against DENV-1, -3, and -4 non-structural proteins following TAK-003 administration. Frequency of IFN-g producing cells as quantified by ELISPOT following DENV-1, -3, or -4 NS1, NS3 or NS5 peptide stimulation at the indicated time points following vaccination. n= 60 for days 0 and 28, n = 54 for day 120. * p < 0.05, **p <0.01, ***p<0.001, ****p<0.0001 Paired 2-tailed T-Test. Bars indicate population mean +/- SEM.

## Slide 3
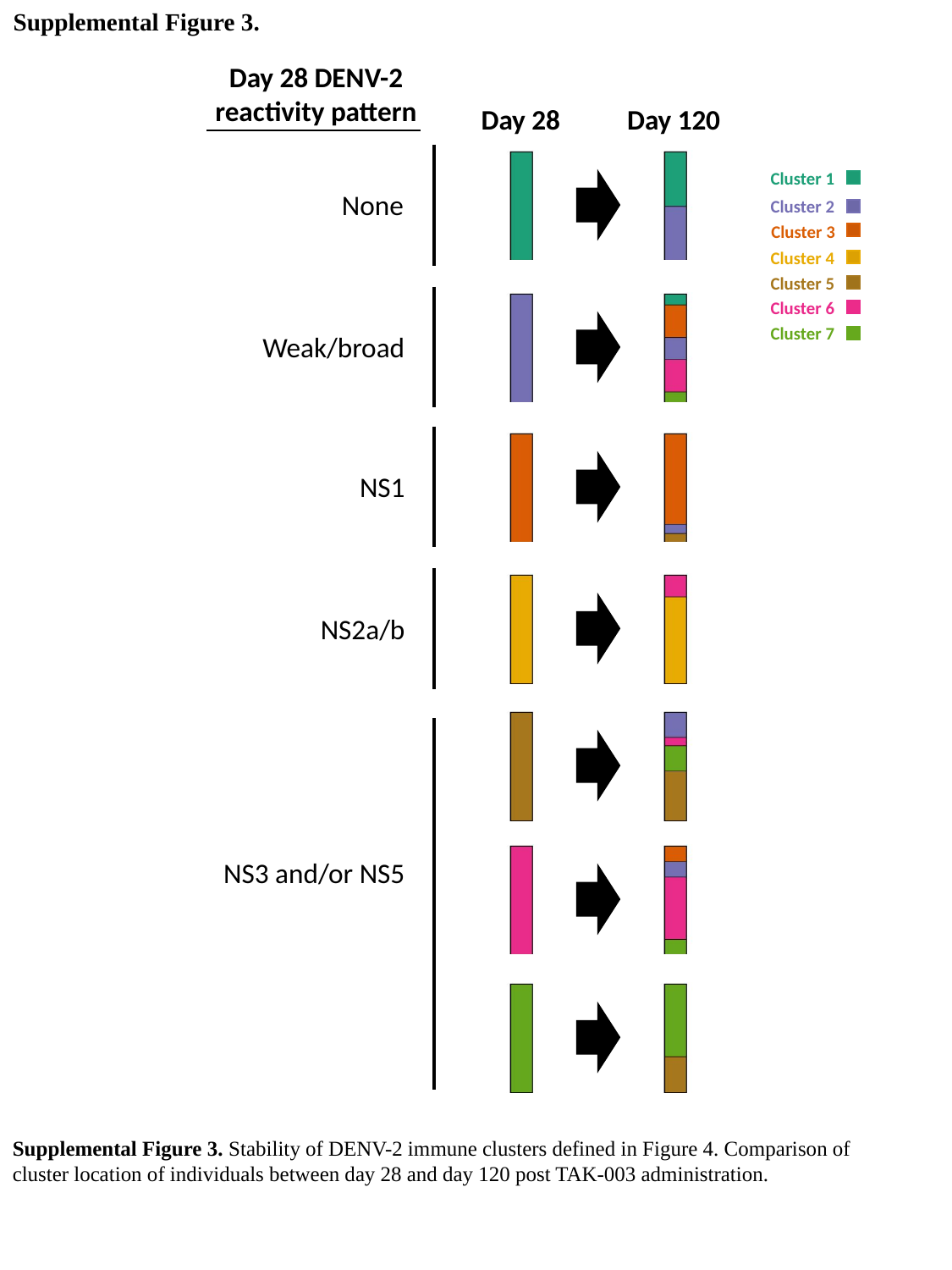

Supplemental Figure 3.
Day 28 DENV-2 reactivity pattern
Day 28
Day 120
Cluster 1
None
Cluster 2
Cluster 3
Cluster 4
Cluster 5
Cluster 6
Cluster 7
Weak/broad
NS1
NS2a/b
NS3 and/or NS5
Supplemental Figure 3. Stability of DENV-2 immune clusters defined in Figure 4. Comparison of cluster location of individuals between day 28 and day 120 post TAK-003 administration.

## Slide 4
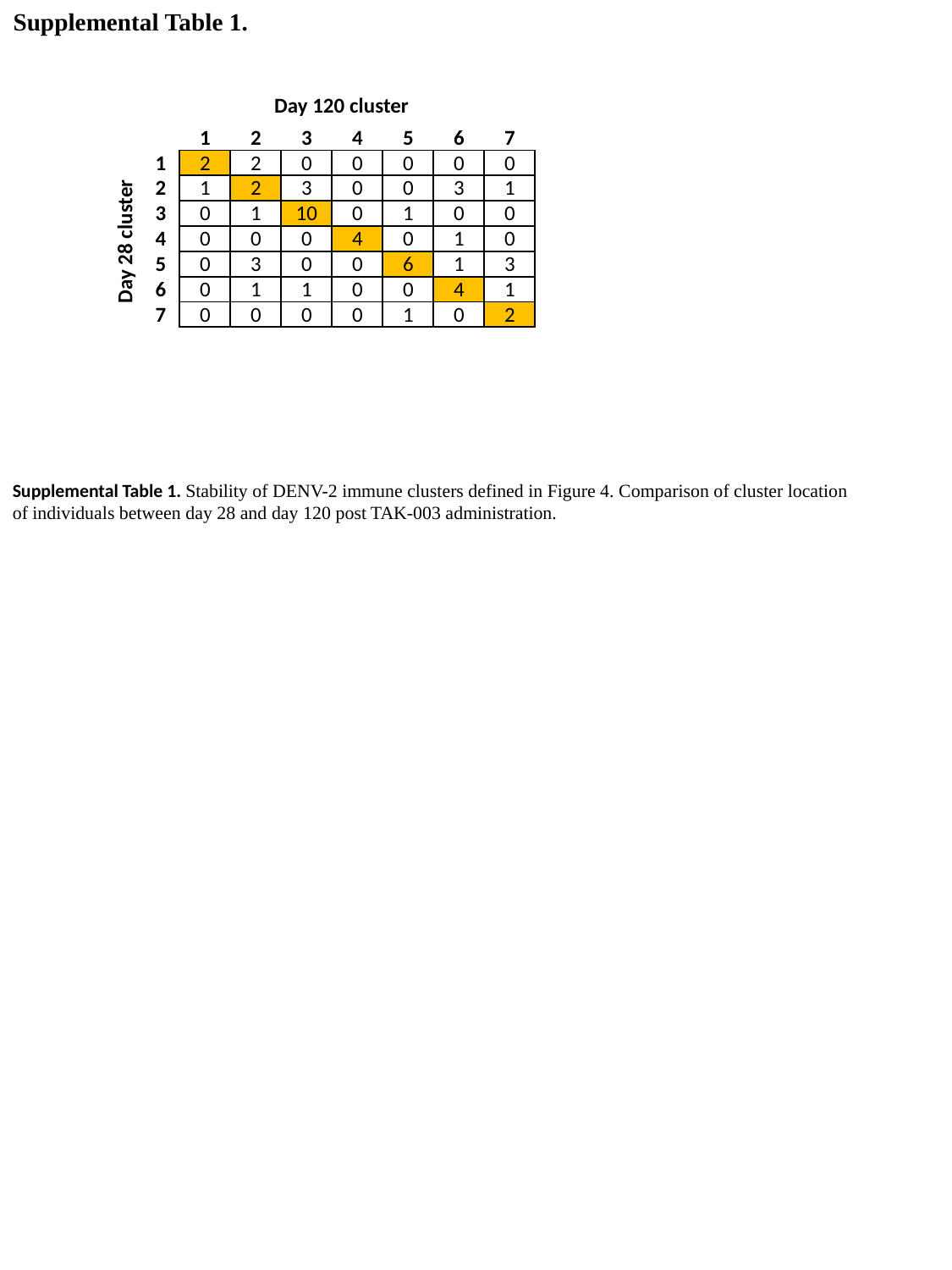

Supplemental Table 1.
Day 120 cluster
| | 1 | 2 | 3 | 4 | 5 | 6 | 7 |
| --- | --- | --- | --- | --- | --- | --- | --- |
| 1 | 2 | 2 | 0 | 0 | 0 | 0 | 0 |
| 2 | 1 | 2 | 3 | 0 | 0 | 3 | 1 |
| 3 | 0 | 1 | 10 | 0 | 1 | 0 | 0 |
| 4 | 0 | 0 | 0 | 4 | 0 | 1 | 0 |
| 5 | 0 | 3 | 0 | 0 | 6 | 1 | 3 |
| 6 | 0 | 1 | 1 | 0 | 0 | 4 | 1 |
| 7 | 0 | 0 | 0 | 0 | 1 | 0 | 2 |
Day 28 cluster
Supplemental Table 1. Stability of DENV-2 immune clusters defined in Figure 4. Comparison of cluster location of individuals between day 28 and day 120 post TAK-003 administration.

## Slide 5
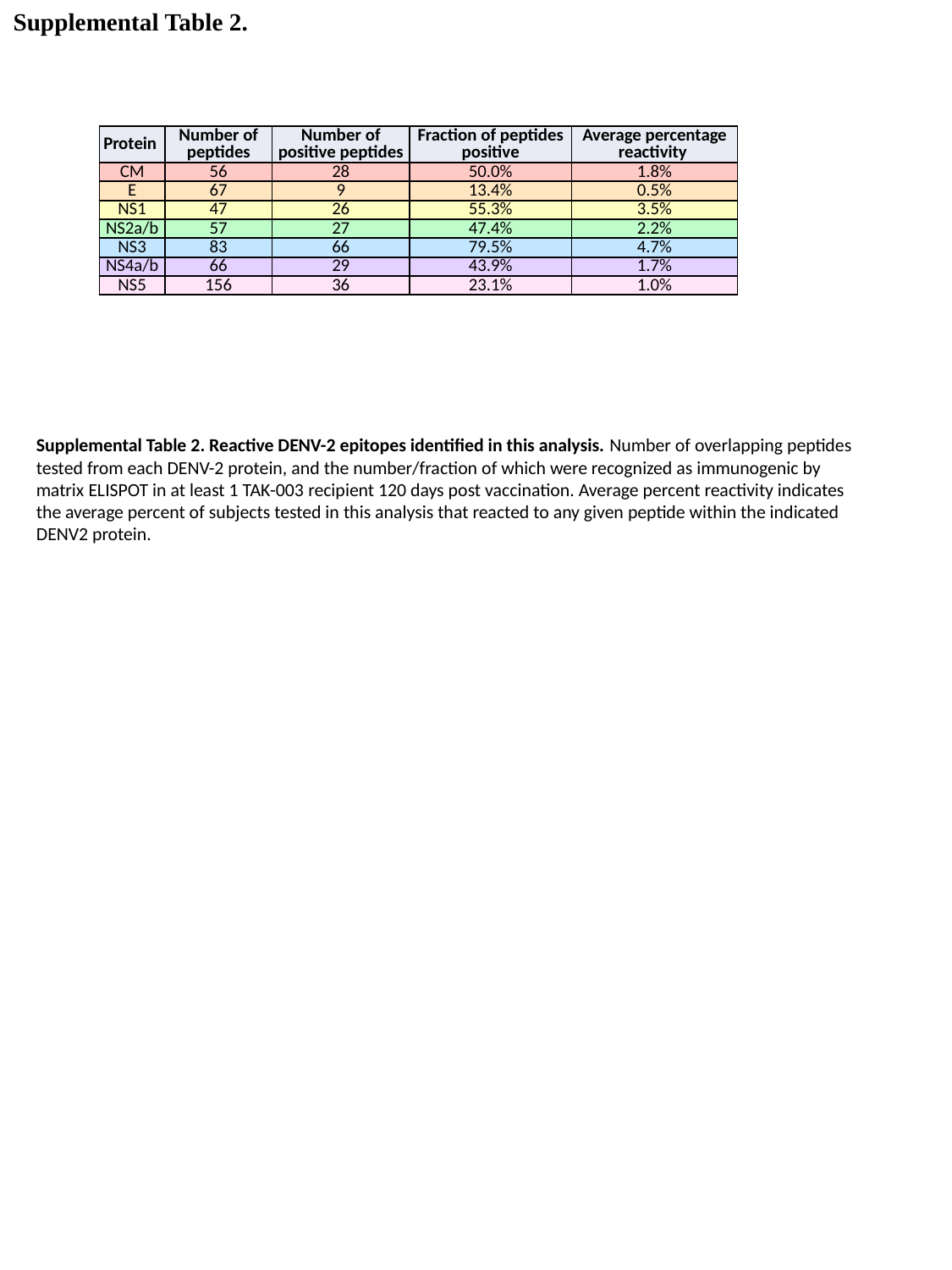

Supplemental Table 2.
| Protein | Number of peptides | Number of positive peptides | Fraction of peptides positive | Average percentage reactivity |
| --- | --- | --- | --- | --- |
| CM | 56 | 28 | 50.0% | 1.8% |
| E | 67 | 9 | 13.4% | 0.5% |
| NS1 | 47 | 26 | 55.3% | 3.5% |
| NS2a/b | 57 | 27 | 47.4% | 2.2% |
| NS3 | 83 | 66 | 79.5% | 4.7% |
| NS4a/b | 66 | 29 | 43.9% | 1.7% |
| NS5 | 156 | 36 | 23.1% | 1.0% |
Supplemental Table 2. Reactive DENV-2 epitopes identified in this analysis. Number of overlapping peptides tested from each DENV-2 protein, and the number/fraction of which were recognized as immunogenic by matrix ELISPOT in at least 1 TAK-003 recipient 120 days post vaccination. Average percent reactivity indicates the average percent of subjects tested in this analysis that reacted to any given peptide within the indicated DENV2 protein.

## Slide 6
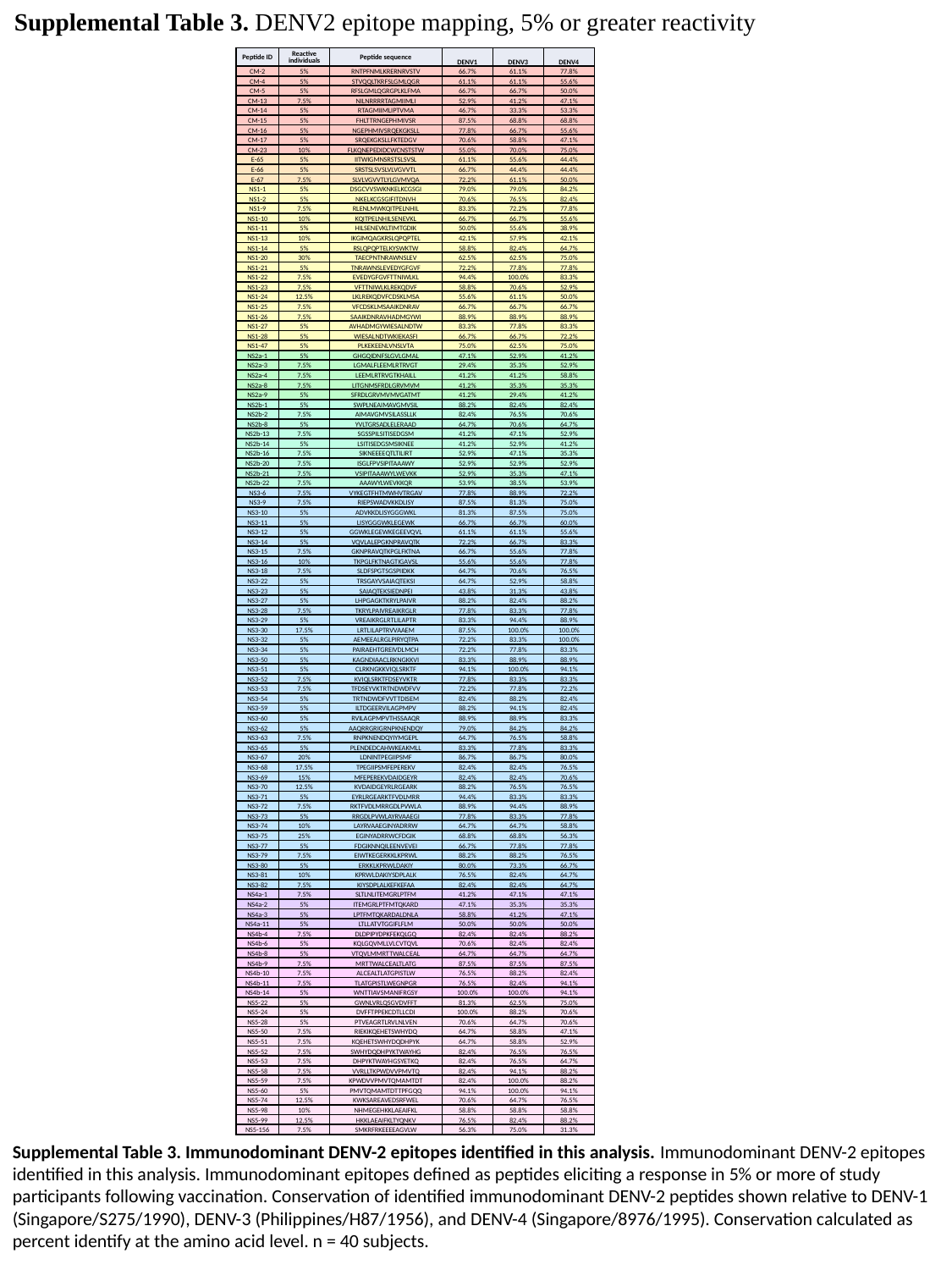

Supplemental Table 3. DENV2 epitope mapping, 5% or greater reactivity
| Peptide ID | Reactive individuals | Peptide sequence | DENV1 | DENV3 | DENV4 |
| --- | --- | --- | --- | --- | --- |
| CM-2 | 5% | RNTPFNMLKRERNRVSTV | 66.7% | 61.1% | 77.8% |
| CM-4 | 5% | STVQQLTKRFSLGMLQGR | 61.1% | 61.1% | 55.6% |
| CM-5 | 5% | RFSLGMLQGRGPLKLFMA | 66.7% | 66.7% | 50.0% |
| CM-13 | 7.5% | NILNRRRRTAGMIIMLI | 52.9% | 41.2% | 47.1% |
| CM-14 | 5% | RTAGMIIMLIPTVMA | 46.7% | 33.3% | 53.3% |
| CM-15 | 5% | FHLTTRNGEPHMIVSR | 87.5% | 68.8% | 68.8% |
| CM-16 | 5% | NGEPHMIVSRQEKGKSLL | 77.8% | 66.7% | 55.6% |
| CM-17 | 5% | SRQEKGKSLLFKTEDGV | 70.6% | 58.8% | 47.1% |
| CM-23 | 10% | FLKQNEPEDIDCWCNSTSTW | 55.0% | 70.0% | 75.0% |
| E-65 | 5% | IITWIGMNSRSTSLSVSL | 61.1% | 55.6% | 44.4% |
| E-66 | 5% | SRSTSLSVSLVLVGVVTL | 66.7% | 44.4% | 44.4% |
| E-67 | 7.5% | SLVLVGVVTLYLGVMVQA | 72.2% | 61.1% | 50.0% |
| NS1-1 | 5% | DSGCVVSWKNKELKCGSGI | 79.0% | 79.0% | 84.2% |
| NS1-2 | 5% | NKELKCGSGIFITDNVH | 70.6% | 76.5% | 82.4% |
| NS1-9 | 7.5% | RLENLMWKQITPELNHIL | 83.3% | 72.2% | 77.8% |
| NS1-10 | 10% | KQITPELNHILSENEVKL | 66.7% | 66.7% | 55.6% |
| NS1-11 | 5% | HILSENEVKLTIMTGDIK | 50.0% | 55.6% | 38.9% |
| NS1-13 | 10% | IKGIMQAGKRSLQPQPTEL | 42.1% | 57.9% | 42.1% |
| NS1-14 | 5% | RSLQPQPTELKYSWKTW | 58.8% | 82.4% | 64.7% |
| NS1-20 | 30% | TAECPNTNRAWNSLEV | 62.5% | 62.5% | 75.0% |
| NS1-21 | 5% | TNRAWNSLEVEDYGFGVF | 72.2% | 77.8% | 77.8% |
| NS1-22 | 7.5% | EVEDYGFGVFTTNIWLKL | 94.4% | 100.0% | 83.3% |
| NS1-23 | 7.5% | VFTTNIWLKLREKQDVF | 58.8% | 70.6% | 52.9% |
| NS1-24 | 12.5% | LKLREKQDVFCDSKLMSA | 55.6% | 61.1% | 50.0% |
| NS1-25 | 7.5% | VFCDSKLMSAAIKDNRAV | 66.7% | 66.7% | 66.7% |
| NS1-26 | 7.5% | SAAIKDNRAVHADMGYWI | 88.9% | 88.9% | 88.9% |
| NS1-27 | 5% | AVHADMGYWIESALNDTW | 83.3% | 77.8% | 83.3% |
| NS1-28 | 5% | WIESALNDTWKIEKASFI | 66.7% | 66.7% | 72.2% |
| NS1-47 | 5% | PLKEKEENLVNSLVTA | 75.0% | 62.5% | 75.0% |
| NS2a-1 | 5% | GHGQIDNFSLGVLGMAL | 47.1% | 52.9% | 41.2% |
| NS2a-3 | 7.5% | LGMALFLEEMLRTRVGT | 29.4% | 35.3% | 52.9% |
| NS2a-4 | 7.5% | LEEMLRTRVGTKHAILL | 41.2% | 41.2% | 58.8% |
| NS2a-8 | 7.5% | LITGNMSFRDLGRVMVM | 41.2% | 35.3% | 35.3% |
| NS2a-9 | 5% | SFRDLGRVMVMVGATMT | 41.2% | 29.4% | 41.2% |
| NS2b-1 | 5% | SWPLNEAIMAVGMVSIL | 88.2% | 82.4% | 82.4% |
| NS2b-2 | 7.5% | AIMAVGMVSILASSLLK | 82.4% | 76.5% | 70.6% |
| NS2b-8 | 5% | YVLTGRSADLELERAAD | 64.7% | 70.6% | 64.7% |
| NS2b-13 | 7.5% | SGSSPILSITISEDGSM | 41.2% | 47.1% | 52.9% |
| NS2b-14 | 5% | LSITISEDGSMSIKNEE | 41.2% | 52.9% | 41.2% |
| NS2b-16 | 7.5% | SIKNEEEEQTLTILIRT | 52.9% | 47.1% | 35.3% |
| NS2b-20 | 7.5% | ISGLFPVSIPITAAAWY | 52.9% | 52.9% | 52.9% |
| NS2b-21 | 7.5% | VSIPITAAAWYLWEVKK | 52.9% | 35.3% | 47.1% |
| NS2b-22 | 7.5% | AAAWYLWEVKKQR | 53.9% | 38.5% | 53.9% |
| NS3-6 | 7.5% | VYKEGTFHTMWHVTRGAV | 77.8% | 88.9% | 72.2% |
| NS3-9 | 7.5% | RIEPSWADVKKDLISY | 87.5% | 81.3% | 75.0% |
| NS3-10 | 5% | ADVKKDLISYGGGWKL | 81.3% | 87.5% | 75.0% |
| NS3-11 | 5% | LISYGGGWKLEGEWK | 66.7% | 66.7% | 60.0% |
| NS3-12 | 5% | GGWKLEGEWKEGEEVQVL | 61.1% | 61.1% | 55.6% |
| NS3-14 | 5% | VQVLALEPGKNPRAVQTK | 72.2% | 66.7% | 83.3% |
| NS3-15 | 7.5% | GKNPRAVQTKPGLFKTNA | 66.7% | 55.6% | 77.8% |
| NS3-16 | 10% | TKPGLFKTNAGTIGAVSL | 55.6% | 55.6% | 77.8% |
| NS3-18 | 7.5% | SLDFSPGTSGSPIIDKK | 64.7% | 70.6% | 76.5% |
| NS3-22 | 5% | TRSGAYVSAIAQTEKSI | 64.7% | 52.9% | 58.8% |
| NS3-23 | 5% | SAIAQTEKSIEDNPEI | 43.8% | 31.3% | 43.8% |
| NS3-27 | 5% | LHPGAGKTKRYLPAIVR | 88.2% | 82.4% | 88.2% |
| NS3-28 | 7.5% | TKRYLPAIVREAIKRGLR | 77.8% | 83.3% | 77.8% |
| NS3-29 | 5% | VREAIKRGLRTLILAPTR | 83.3% | 94.4% | 88.9% |
| NS3-30 | 17.5% | LRTLILAPTRVVAAEM | 87.5% | 100.0% | 100.0% |
| NS3-32 | 5% | AEMEEALRGLPIRYQTPA | 72.2% | 83.3% | 100.0% |
| NS3-34 | 5% | PAIRAEHTGREIVDLMCH | 72.2% | 77.8% | 83.3% |
| NS3-50 | 5% | KAGNDIAACLRKNGKKVI | 83.3% | 88.9% | 88.9% |
| NS3-51 | 5% | CLRKNGKKVIQLSRKTF | 94.1% | 100.0% | 94.1% |
| NS3-52 | 7.5% | KVIQLSRKTFDSEYVKTR | 77.8% | 83.3% | 83.3% |
| NS3-53 | 7.5% | TFDSEYVKTRTNDWDFVV | 72.2% | 77.8% | 72.2% |
| NS3-54 | 5% | TRTNDWDFVVTTDISEM | 82.4% | 88.2% | 82.4% |
| NS3-59 | 5% | ILTDGEERVILAGPMPV | 88.2% | 94.1% | 82.4% |
| NS3-60 | 5% | RVILAGPMPVTHSSAAQR | 88.9% | 88.9% | 83.3% |
| NS3-62 | 5% | AAQRRGRIGRNPKNENDQY | 79.0% | 84.2% | 84.2% |
| NS3-63 | 7.5% | RNPKNENDQYIYMGEPL | 64.7% | 76.5% | 58.8% |
| NS3-65 | 5% | PLENDEDCAHWKEAKMLL | 83.3% | 77.8% | 83.3% |
| NS3-67 | 20% | LDNINTPEGIIPSMF | 86.7% | 86.7% | 80.0% |
| NS3-68 | 17.5% | TPEGIIPSMFEPEREKV | 82.4% | 82.4% | 76.5% |
| NS3-69 | 15% | MFEPEREKVDAIDGEYR | 82.4% | 82.4% | 70.6% |
| NS3-70 | 12.5% | KVDAIDGEYRLRGEARK | 88.2% | 76.5% | 76.5% |
| NS3-71 | 5% | EYRLRGEARKTFVDLMRR | 94.4% | 83.3% | 83.3% |
| NS3-72 | 7.5% | RKTFVDLMRRGDLPVWLA | 88.9% | 94.4% | 88.9% |
| NS3-73 | 5% | RRGDLPVWLAYRVAAEGI | 77.8% | 83.3% | 77.8% |
| NS3-74 | 10% | LAYRVAAEGINYADRRW | 64.7% | 64.7% | 58.8% |
| NS3-75 | 25% | EGINYADRRWCFDGIK | 68.8% | 68.8% | 56.3% |
| NS3-77 | 5% | FDGIKNNQILEENVEVEI | 66.7% | 77.8% | 77.8% |
| NS3-79 | 7.5% | EIWTKEGERKKLKPRWL | 88.2% | 88.2% | 76.5% |
| NS3-80 | 5% | ERKKLKPRWLDAKIY | 80.0% | 73.3% | 66.7% |
| NS3-81 | 10% | KPRWLDAKIYSDPLALK | 76.5% | 82.4% | 64.7% |
| NS3-82 | 7.5% | KIYSDPLALKEFKEFAA | 82.4% | 82.4% | 64.7% |
| NS4a-1 | 7.5% | SLTLNLITEMGRLPTFM | 41.2% | 47.1% | 47.1% |
| NS4a-2 | 5% | ITEMGRLPTFMTQKARD | 47.1% | 35.3% | 35.3% |
| NS4a-3 | 5% | LPTFMTQKARDALDNLA | 58.8% | 41.2% | 47.1% |
| NS4a-11 | 5% | LTLLATVTGGIFLFLM | 50.0% | 50.0% | 50.0% |
| NS4b-4 | 7.5% | DLDPIPYDPKFEKQLGQ | 82.4% | 82.4% | 88.2% |
| NS4b-6 | 5% | KQLGQVMLLVLCVTQVL | 70.6% | 82.4% | 82.4% |
| NS4b-8 | 5% | VTQVLMMRTTWALCEAL | 64.7% | 64.7% | 64.7% |
| NS4b-9 | 7.5% | MRTTWALCEALTLATG | 87.5% | 87.5% | 87.5% |
| NS4b-10 | 7.5% | ALCEALTLATGPISTLW | 76.5% | 88.2% | 82.4% |
| NS4b-11 | 7.5% | TLATGPISTLWEGNPGR | 76.5% | 82.4% | 94.1% |
| NS4b-14 | 5% | WNTTIAVSMANIFRGSY | 100.0% | 100.0% | 94.1% |
| NS5-22 | 5% | GWNLVRLQSGVDVFFT | 81.3% | 62.5% | 75.0% |
| NS5-24 | 5% | DVFFTPPEKCDTLLCDI | 100.0% | 88.2% | 70.6% |
| NS5-28 | 5% | PTVEAGRTLRVLNLVEN | 70.6% | 64.7% | 70.6% |
| NS5-50 | 7.5% | RIEKIKQEHETSWHYDQ | 64.7% | 58.8% | 47.1% |
| NS5-51 | 7.5% | KQEHETSWHYDQDHPYK | 64.7% | 58.8% | 52.9% |
| NS5-52 | 7.5% | SWHYDQDHPYKTWAYHG | 82.4% | 76.5% | 76.5% |
| NS5-53 | 7.5% | DHPYKTWAYHGSYETKQ | 82.4% | 76.5% | 64.7% |
| NS5-58 | 7.5% | VVRLLTKPWDVVPMVTQ | 82.4% | 94.1% | 88.2% |
| NS5-59 | 7.5% | KPWDVVPMVTQMAMTDT | 82.4% | 100.0% | 88.2% |
| NS5-60 | 5% | PMVTQMAMTDTTPFGQQ | 94.1% | 100.0% | 94.1% |
| NS5-74 | 12.5% | KWKSAREAVEDSRFWEL | 70.6% | 64.7% | 76.5% |
| NS5-98 | 10% | NHMEGEHKKLAEAIFKL | 58.8% | 58.8% | 58.8% |
| NS5-99 | 12.5% | HKKLAEAIFKLTYQNKV | 76.5% | 82.4% | 88.2% |
| NS5-156 | 7.5% | SMKRFRKEEEEAGVLW | 56.3% | 75.0% | 31.3% |
Supplemental Table 3. Immunodominant DENV-2 epitopes identified in this analysis. Immunodominant DENV-2 epitopes identified in this analysis. Immunodominant epitopes defined as peptides eliciting a response in 5% or more of study participants following vaccination. Conservation of identified immunodominant DENV-2 peptides shown relative to DENV-1 (Singapore/S275/1990), DENV-3 (Philippines/H87/1956), and DENV-4 (Singapore/8976/1995). Conservation calculated as percent identify at the amino acid level. n = 40 subjects.

## Slide 7
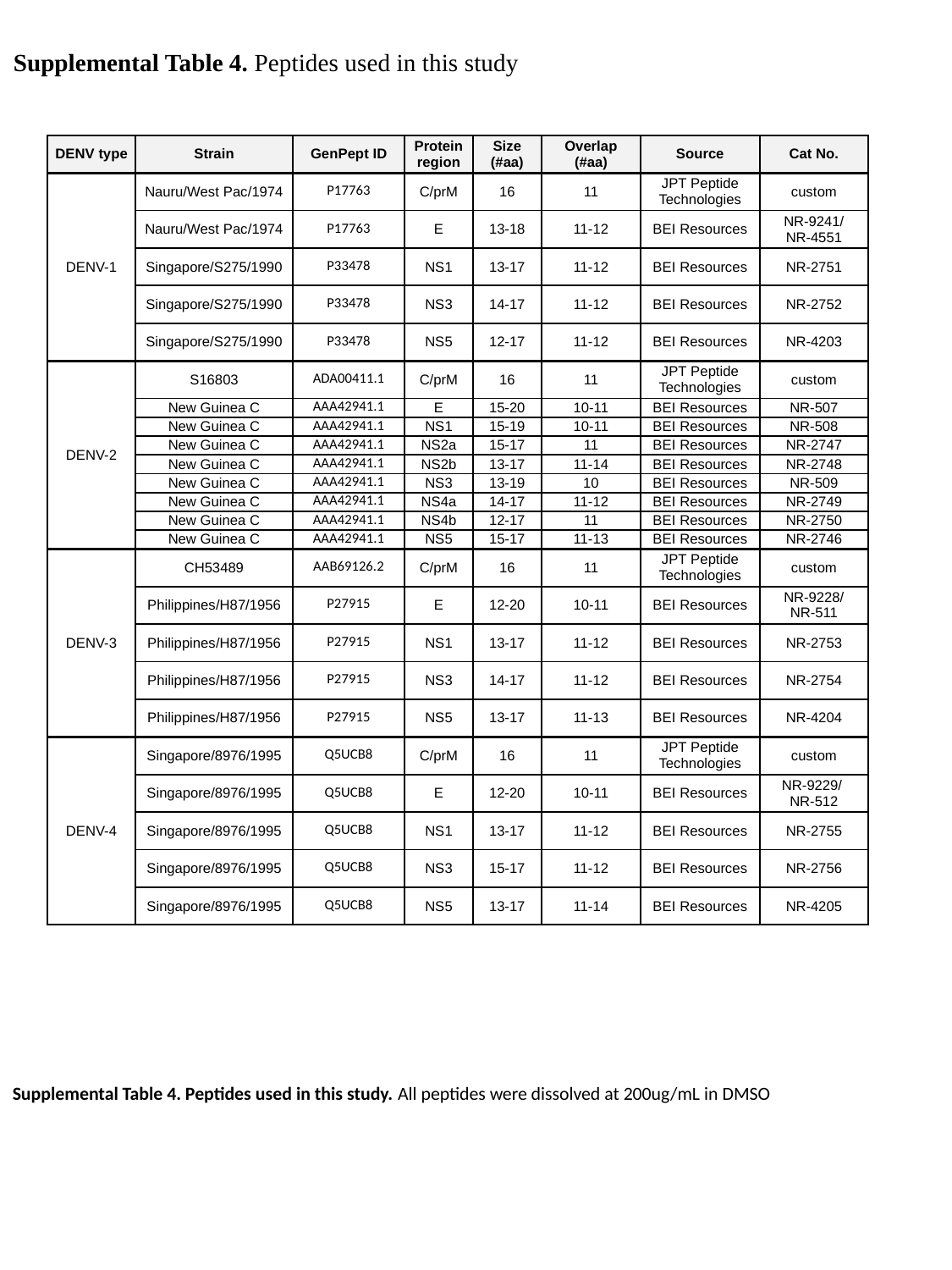

Supplemental Table 4. Peptides used in this study
| DENV type | Strain | GenPept ID | Protein region | Size (#aa) | Overlap (#aa) | Source | Cat No. |
| --- | --- | --- | --- | --- | --- | --- | --- |
| DENV-1 | Nauru/West Pac/1974 | P17763 | C/prM | 16 | 11 | JPT Peptide Technologies | custom |
| | Nauru/West Pac/1974 | P17763 | E | 13-18 | 11-12 | BEI Resources | NR-9241/ NR-4551 |
| | Singapore/S275/1990 | P33478 | NS1 | 13-17 | 11-12 | BEI Resources | NR-2751 |
| | Singapore/S275/1990 | P33478 | NS3 | 14-17 | 11-12 | BEI Resources | NR-2752 |
| | Singapore/S275/1990 | P33478 | NS5 | 12-17 | 11-12 | BEI Resources | NR-4203 |
| DENV-2 | S16803 | ADA00411.1 | C/prM | 16 | 11 | JPT Peptide Technologies | custom |
| | New Guinea C | AAA42941.1 | E | 15-20 | 10-11 | BEI Resources | NR-507 |
| | New Guinea C | AAA42941.1 | NS1 | 15-19 | 10-11 | BEI Resources | NR-508 |
| | New Guinea C | AAA42941.1 | NS2a | 15-17 | 11 | BEI Resources | NR-2747 |
| | New Guinea C | AAA42941.1 | NS2b | 13-17 | 11-14 | BEI Resources | NR-2748 |
| | New Guinea C | AAA42941.1 | NS3 | 13-19 | 10 | BEI Resources | NR-509 |
| | New Guinea C | AAA42941.1 | NS4a | 14-17 | 11-12 | BEI Resources | NR-2749 |
| | New Guinea C | AAA42941.1 | NS4b | 12-17 | 11 | BEI Resources | NR-2750 |
| | New Guinea C | AAA42941.1 | NS5 | 15-17 | 11-13 | BEI Resources | NR-2746 |
| DENV-3 | CH53489 | AAB69126.2 | C/prM | 16 | 11 | JPT Peptide Technologies | custom |
| | Philippines/H87/1956 | P27915 | E | 12-20 | 10-11 | BEI Resources | NR-9228/ NR-511 |
| | Philippines/H87/1956 | P27915 | NS1 | 13-17 | 11-12 | BEI Resources | NR-2753 |
| | Philippines/H87/1956 | P27915 | NS3 | 14-17 | 11-12 | BEI Resources | NR-2754 |
| | Philippines/H87/1956 | P27915 | NS5 | 13-17 | 11-13 | BEI Resources | NR-4204 |
| DENV-4 | Singapore/8976/1995 | Q5UCB8 | C/prM | 16 | 11 | JPT Peptide Technologies | custom |
| | Singapore/8976/1995 | Q5UCB8 | E | 12-20 | 10-11 | BEI Resources | NR-9229/ NR-512 |
| | Singapore/8976/1995 | Q5UCB8 | NS1 | 13-17 | 11-12 | BEI Resources | NR-2755 |
| | Singapore/8976/1995 | Q5UCB8 | NS3 | 15-17 | 11-12 | BEI Resources | NR-2756 |
| | Singapore/8976/1995 | Q5UCB8 | NS5 | 13-17 | 11-14 | BEI Resources | NR-4205 |
Supplemental Table 4. Peptides used in this study. All peptides were dissolved at 200ug/mL in DMSO
